# Influence of the Composition on the Environmental Impact of Soft Ferrites

**DOI:** 10.3390/ma11101789

**Published:** 2018-09-20

**Authors:** Patricia Gómez, Daniel Elduque, Carmelo Pina, Carlos Javierre

**Affiliations:** 1 BSH Electrodomésticos España S. A., Avda. de la Industria, 49, 50016 Zaragoza, Spain; patricia.gomez@bshg.com (P.G.); carmelo.pina@bshg.com (C.P.); 2 i+AITIIP, Department of Mechanical Engineering, EINA; University of Zaragoza, C/María de Luna, 3, 50018 Zaragoza, Spain; carlos.javierre@unizar.es

**Keywords:** LCA, soft ferrites, magnetic materials, MnZn, NiZn, material composition, environmental impact, critical raw materials

## Abstract

The aim of this paper is to analyze the influence of the composition on the environmental impact of the two main types of soft ferrites, allowing scientists and engineers to compare them based not only on cost and properties, but also on an environmental point of view. Iron oxides are the basis of soft ferrites, but these ferrites have a wide range of compositions, using materials such as manganese or nickel, which affect their magnetic properties, but also modify the environmental impact. A Life Cycle Assessment has been carried out for manganese‒zinc (MnZn) and nickel‒zinc (NiZn) soft ferrites, with a Monte Carlo approach to assess multiple compositions. The LCA model was developed with SimaPro 8.4, using the EcoInvent v3.4 life cycle inventory database. Environmental impact values were calculated under the ReCiPe and Carbon Footprint methodologies, obtaining a broad variety of results depending on the composition. The results were also significantly different from the standard EcoInvent ferrite. For the analyzed soft ferrites, the presence of manganese or nickel is a key factor from an environmental perspective, as these materials involve high environmental impacts, and their supply risk has increased during recent years, making them a concern for European manufacturers.

## 1. Introduction

Issues such as pollution and climate change have caused people’s concern about environmental impacts to increase exponentially. At the end of the 20th century, the concept of Ecodesign started, with the aim of prevention during the design stage, instead of correction afterward [1]. Following these ecological trends, enterprises are making efforts to reduce the environmental impact of their products, processes, and waste [2,3]. Standards such as ISO 14006 promote minimal environmental impact on product development [4], and several policies, such as EuP 2005/32/CE and ErP 2009/125/CE, contribute to a green economy and Ecodesign in the European Union [5,6,7,8].

For all these reasons, during the design of a new product, material selection is considered one of the main influences on the entire life cycle, and therefore it is essential to assess the environmental impact of materials accurately, taking into account their actual composition [9,10,11].

One of the primary methodologies to measure environmental impact is Life Cycle Assessment (LCA). LCA evaluates the environmental impact of a product, material, service, or process [12,13,14]. There are many examples of LCA applied to specific materials such as asphalt or concrete road pavement materials [15], wood [16], plastics [17]; or metallic materials such as lead [18], steel [19] a and aluminum [20]. Other authors have focused their studies on methods to reduce the overall consumption of raw materials, especially, of critical raw materials [21].

LCA helps companies evaluate how environmental impact is generated, enabling them to modify and reduce the environmental impact of their products in the design phase, instead of correcting it later [11,22,23]. LCA relies on large Life Cycle Inventory (LCI) databases, such as EcoInvent, which helps to assign each material, process, or transport an environmental impact. The problem with these databases is that they only have a limited amount of datasets, therefore reducing the accuracy of the environmental impact results [9,24]. To properly assess the environmental impact, it is key to consider the exact material composition. Industries, enterprises, and governments are currently using LCA to analyze the environmental impact, and also to assess their material consumption and supply risk [25]. During recent years, the European Union and other governments and associations have developed a list of materials that are considered strategic due to their economic importance and supply risk. Those materials with both high risk and high importance are called critical raw materials [26].

This paper aims to examine and better understand the influence of the composition on the environmental impact of soft ferrite magnetic materials. Ferrites use materials such as manganese or nickel, which are included on those lists.

Ferrites started to be used in the industry in the middle of the 20th century, after they were discovered by Dr. Kato and Dr. Takei in 1930 [27]. The main applications of ferrites were in electronic devices: transformers, anti-electromagnetic filters, or magnetic recording media [28,29,30,31,32,33]. Apart from those applications, nowadays the consumption of ferrites is increasing in other types of electronic devices, such as televisions and radios, thanks to their low cost and mechanical resistance [34]. Although the use of ferrites has mainly been focused on electronic components, they are also used in different applications, for example, in wastewater treatments [35,36,37], as a catalyst to increase the reaction rate of chemical reactions [38], as indicators in magnetic resonance [39], or in hyperthermia treatments [40].

Ferrites are mainly classified according to their chemical formula in: spinel, garnet, hexaferrites, and orthoferrites. Spinel is the most used type, and its chemical formula is MFeO, where M are metallic cations like cobalt [41,42,43], manganese‒zinc alloy [44,45], nickel‒zinc or other metals like iron [46]; and FeO are iron oxides [47]. Their chemical formula and structure gives these compounds different properties, such as high magnetic permeability, high resistivity, or high Curie temperature [48,49]. They are also classified into hard and soft ferrites depending on their resistance to being demagnetized [47]. Hard ferrites are considered excellent magnetic materials or even permanent magnets due to their high resistance to being demagnetized. The second type, soft ferrites, have low coercivity, changing their magnetization easily; therefore, they are excellent magnetic cores for induction fields.

In the case of soft ferrites, the two main types are manganese‒zinc (MnZn) and nickel‒zinc (NiZn). Both have similar properties: low coercivity with high resistivity, low losses, and high permeability. They are commonly used in high-frequency applications, and both types share a common working area for frequencies between 10^−2^ and 1 MHz frequency, as well as an initial permeability of around 10^3^ [47]. This study focuses on comparing these two types of soft ferrites from an environmental point of view, as composition changes involve modifications of the environmental impact. Although iron oxides are usually in higher proportion than metallic cations, the latter generate most of the environmental impact of these compounds, as many of these metallic cation materials (such as manganese or nickel) have an important environmental impact.

Since 2011, every three years the European Union assesses how critical raw materials are for their economy, following a specific methodology that considers criteria such as supply risk and economic importance [50,51,52,53]. In the last report, 27 materials were defined as critical by the EU out of the 78 strategic materials analyzed [54,55]. Manganese, nickel, and zinc, included in the analyzed soft ferrites, were considered as strategic materials in the last 2017 EU report. According to it, manganese has a high supply risk, given that 90% of the EU supply comes from only three countries, and it is of significant economic importance as it is related to the production of steel and other non-steel alloys. Nickel also has high economic importance as it is used in stainless steel and other steel alloys, but its supply is more diversified, therefore presenting lower risk. Finally, zinc is characterized with similar economic importance and supply risk as nickel. It is mainly used for steel and zinc alloys [54].

In this paper, a LCA has been carried out analyzing the EcoInvent ferrite dataset and customizing it with material compositions of manganese‒zinc (MnZn) and nickel‒zinc (NiZn) ferrites, the two major categories of soft ferrites.

## 2. Methods

This research is based on the EcoInvent ferrite dataset, but customized to MnZn and NiZn soft ferrites, which have an extensive range of compositions depending on their amount of zinc, nickel, and manganese oxides.

### 2.1. Functional Unit and System Boundaries

In order to carry out an LCA, the functional unit is defined as the production of 1 kg of soft ferrite. Therefore, all the results are shown on a kilogram basis.

**Figure 1:**
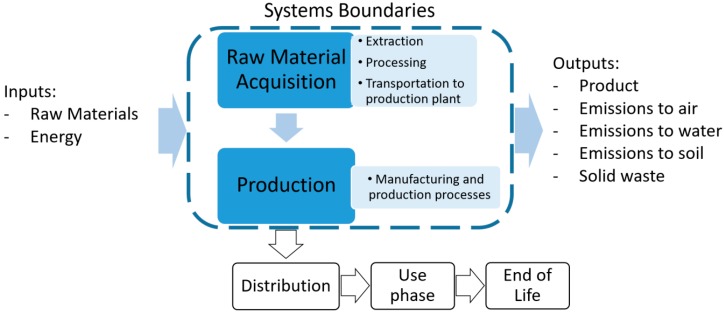
System boundaries.

The LCA has been carried out to assess the environmental impact of a wide range of MnZn and NiZn ferrite compositions. Considering the selected functional unit, a cradle to gate approach has been followed, including raw materials and ferrite production. The first stage includes Raw Material Acquisition (RMA) and its transportation to the factory, whereas the manufacturing stage considers all the related inputs: energy consumption (electricity, natural gas, coal, etc.), and the infrastructure efforts. Distribution to the customer, use phase, and end of life are outside of the boundaries of our consideration, as the aim of this study is to investigate the influence of the variation of the composition. The system boundaries are shown in Figure 1.

Following the EcoInvent methodology, market datasets have been used to consider the transportation processes of raw materials from average providers to a ferrite manufacturing plant [56]. To consider these life cycle stages, the ISO 14040 and 14044 standards have been followed [57,58].

### 2.2. Inventory Data and Assumptions

The software used to develop the LCA model was SimaPro 8.4 [59], with the EcoInvent v3.4 LCI database, one of the most well-known databases, which is developed by the Swiss Center for Life Cycle Inventories [56]. Both are currently the most used tools to evaluate environmental impact in the LCA scientific community.

This LCA aims to analyze the influence of the composition on the environmental impact of soft ferrites. The EcoInvent dataset “Ferrite production {GLO}” has been used as a reference, following the EcoInvent methodology and assumptions, but customizing it to include compositions of MnZn and NiZn soft ferrites. Table 1 shows the selected EcoInvent datasets for the ferrite materials.

In order to evaluate the environmental impact, the ReCiPe EndPoint (H/A) and IPCC 2013 Carbon footprint GWP100a methodologies were used [59]. ReCiPe considers a wide range of environmental impact categories, and its EndPoint approach makes the results easier to analyze from an engineering point of view, allowing us to perform easier comparisons between different materials. On the other hand, the IPCC 2013 Carbon footprint GWP100a establishes the emissions generated by a product expressed as Carbon Dioxide equivalents, focusing only on one environmental impact category, that is, Climate Change, which has special social relevance.

### 2.3. Life Cycle Inventory

After establishing the LCA framework, the Life Cycle Inventories have to be defined for both MnZn and NiZn soft ferrites. 

Table 2 shows the minimum and maximum molar percentages of MnZn [60]. These percentages have to be transformed into mass percentages (Table 3) in order to quantify the environmental impact of 1 kg of soft ferrite material.

Table 4 shows the minimum and maximum molar percentages of NiZn [60]. As in the previous ones, these percentages have been transformed into mass percentages (Table 5).

The percentages composition range shown in Table 3 and Table 5 establish all the possible composition combinations for soft ferrites, based on the molar composition shown in Table 2 and Table 4.

Table 6 shows the inventory for the production processes of 1 kg of soft ferrite. These values are obtained from the EcoInvent data.

As previously explained, market datasets have been used to consider the transportation processes of raw materials from average providers to a ferrite manufacturing plant. Table 7 shows the used EcoInvent data.

**Figure 2:**
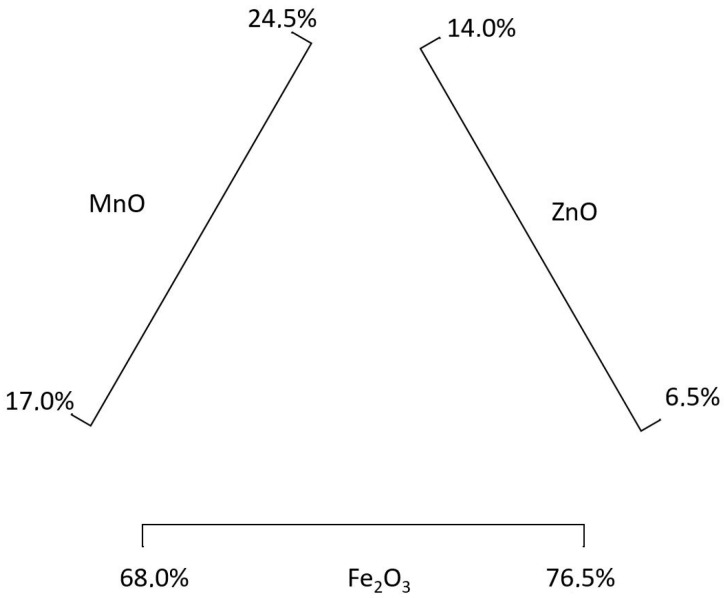
MnZn soft ferrite composition diagram.

**Figure 3:**
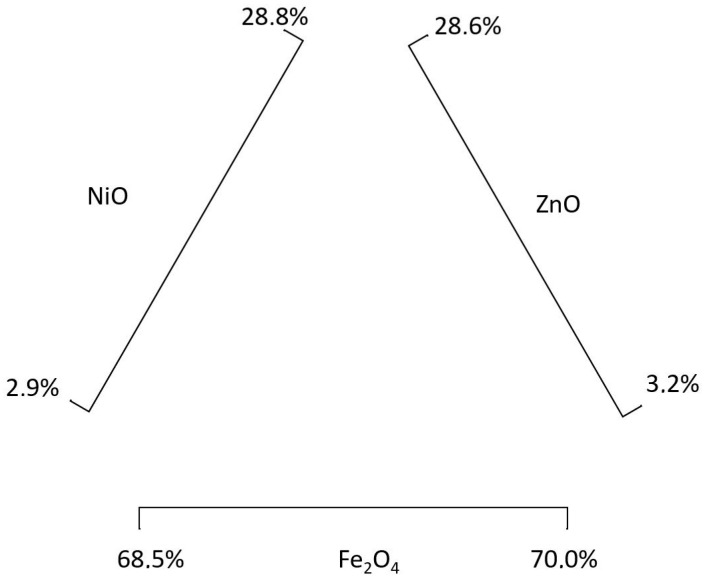
NiZn soft ferrite composition diagram.

Figure 2 shows the composition diagram for MnZn ferrites, and Figure 3 for NiZn ferrites. As there are multiple composition combinations, for each ferrite a wide variety of compositions are going to be calculated. For this research, a Monte Carlo analysis is carried out in order to analyze the environmental impact of an extensive range of soft ferrites compositions. In order to do that, the input values to obtain 1 kg of soft ferrite have been modified for each of the materials, considering a uniform distribution. This means that any value within the composition ranges (Table 3 and Table 5) is as probable as the others. The rest of the inventory values of this study have been considered constant in this Monte Carlo approach.

The Monte Carlo analysis first provides the calculated material inputs, and then, the environmental impact results are calculated. These input values have to be normalized to 1 kg of soft ferrite, which is the functional unit, and checked to make sure that they comply with the composition ranges (e.g., 765 g of Fe_2_O_3_ + 245 g of MnO + 140 g of ZnO can be provided by the Monte Carlo analysis; but when normalized to 1000 g of ferrite, the Fe_2_O_3_ content is 648.3 g, 64.8%, and therefore outside of the defined range).

## 3. Results and Discussion

**Figure 4:**
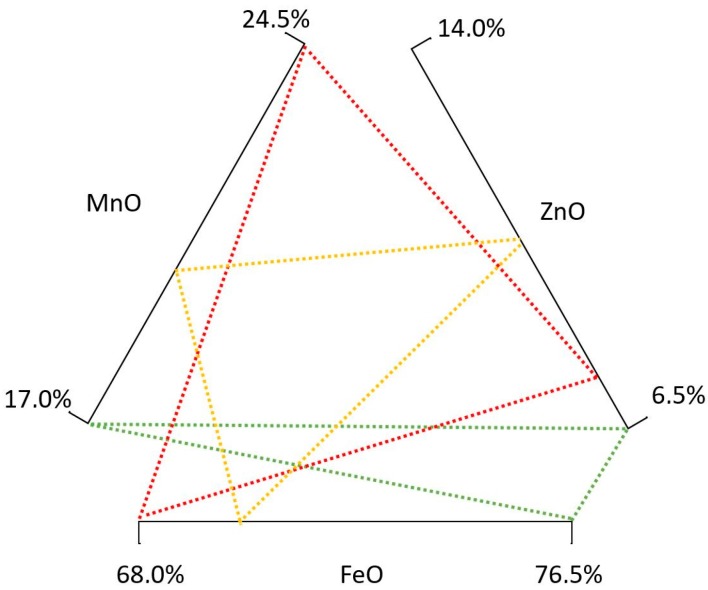
MnZn soft ferrite environmental impact diagram.

**Figure 5:**
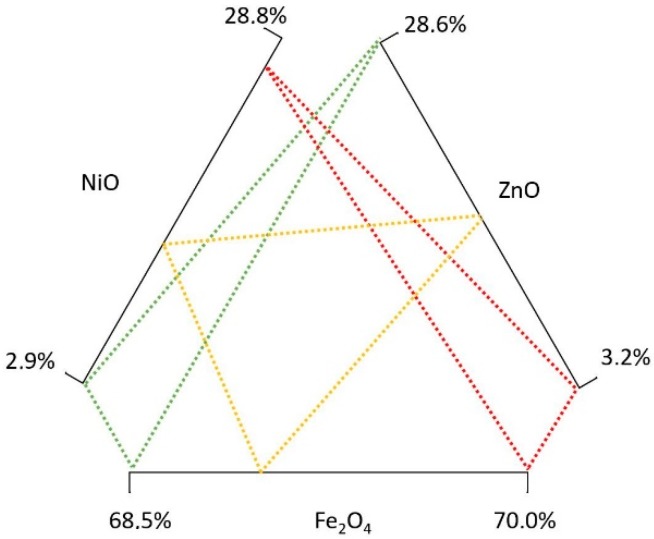
NiZn soft ferrite environmental impact diagram.

In this section, the results of the LCA study are given. Using the composition ranges of Table 3 and Table 5, a Monte Carlo analysis was carried out to assess the environmental impacts of soft ferrites that complied with the respective composition range. In total, 67 MnZn and 67 NiZn soft ferrites were analyzed. Table 8 shows the main environmental impact results of MnZn ferrites, indicating maximum, average, and minimum environmental impact combinations in Figure 4, whereas the results for NiZn are shown in Table 9 and represented in Figure 5. These results consider all the stages within the system boundaries.

For MnZn ferrites, impacts range from 1572 mPt/kg up to 2224 mPt/kg. Whereas, using the Carbon Footprint methodology, MnZn ferrites range from 1.02 up to 1.29 Kg CO_2_ eq.

Table 8 and Figure 4 show that, under the ReCiPe and Carbon Footprint methodologies, the MnZn ferrite, with 6.5% ZnO and 17% MnO, has the lowest environmental impact (green lines in Figure 4). The composition with 7.5% ZnO and 24.5% MnO (red lines in Figure 4) creates the highest environmental impact. Finally, the yellow lines show the average composition. These differences are largely created by the content of manganese, as this material involves high environmental impact values (8769 mPt/kg in the ReCiPe, and 3.59 kg CO_2_ eq. per kg in the Carbon Footprint methodology).

Manganese is also included in the 2017 EU strategic materials list due to its high economic importance in the EU industry, and its supply risk, but it is not currently considered among the 27 materials from the critical list. Analyzing the European Union data on Critical Raw Materials, we can see that the economic importance of manganese has decreased in the last three years, whereas the supply risk indicator has doubled, making it an import issue for EU manufacturers.

The same analysis approach has been followed for NiZn ferrites. Figure 5 and Table 9 show the results using the ReCiPe and Carbon Footprint methodologies. These impacts range from 272 mPt/kg up to 1682 mPt/kg. Using the Carbon Footprint methodology, NiZn ferrites vary from 0.94 up to 3.46 kg CO_2_ eq. per kg.

The NiZn ferrite that creates the lowest environmental impact (green lines in Figure 5) has 2.9% of NiO and 28.6% of ZnO. In contrast, a content of 26.8% NiO and 3.2% ZnO, generates the highest environmental impact per kg (red lines in Figure 5). These data show that the main differences are created by the use of nickel in the composition. Nickel has a high environmental impact (6006 mPt/kg in the ReCiPe, and 11.5 kg CO_2_ eq. per kg in the Carbon Footprint methodology), and is also considered a strategic material, but, currently, it is not in the critical list. Analyzing the European Union data on Critical Raw Materials, although the economic importance has slightly decreased in the last three years, its supply risk has increased.

In order to further analyze the environmental impact of soft ferrites, the following figure allows us to quantify the percentages of the environmental impact, both in ReCiPe and Carbon Footprint, that are caused by the RMA of each material, and which are related to the production processes at the manufacturing plant. This analysis is going to be performed for the average compositions of MnZn and NiZn, as they are the most representative for each ferrite type and, finally, for the EcoInvent ferrite dataset, which is used as a benchmark.

**Figure 6:**
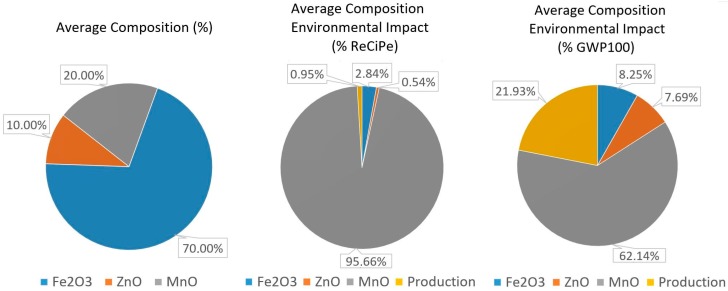
MnZn soft ferrite with average composition environmental impact in the ReCiPe and Carbon Footprint methodologies.

Focusing on MnZn ferrites, the average composition in MnZn ferrite is the one with 70% of Fe_2_O_3_, 20% MnO, and 10% of ZnO (Figure 6). When analyzing the environmental impact under the ReCiPe methodology, the presence of manganese oxide generates almost 95.7% of the overall environmental impact. Iron oxide creates 2.84% of the impact, whereas production processes only account for 0.95%. Under the Carbon Footprint methodology, the content of MnO generates more than 62.1% of the total environmental impact, followed by production processes, which create 21.9% of the environmental impact. In both methodologies, the presence of zinc oxide produces the lowest environmental impact due to its low composition percentages, and also its low environmental impacts, with 99 mPt/kg in the ReCiPe and, almost 0.89 kg CO_2_ eq. per kg, with the Carbon Footprint methodology.

**Figure 7:**
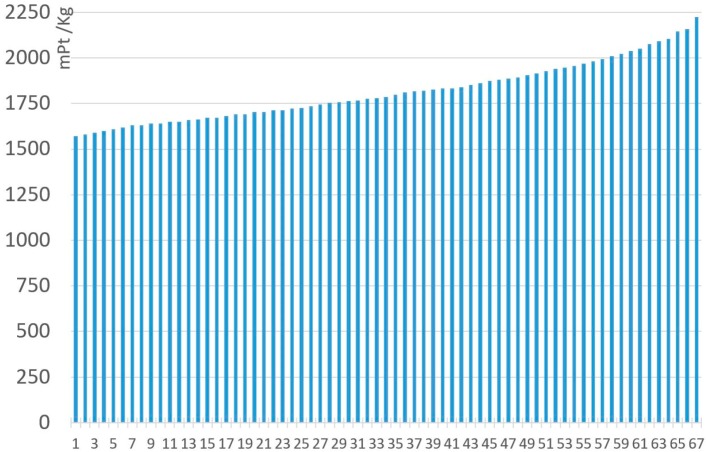
Environmental impact (RMA + Production). of 1 kg MnZn for the 67 ferrites, under the ReCiPe methodology.

**Figure 8:**
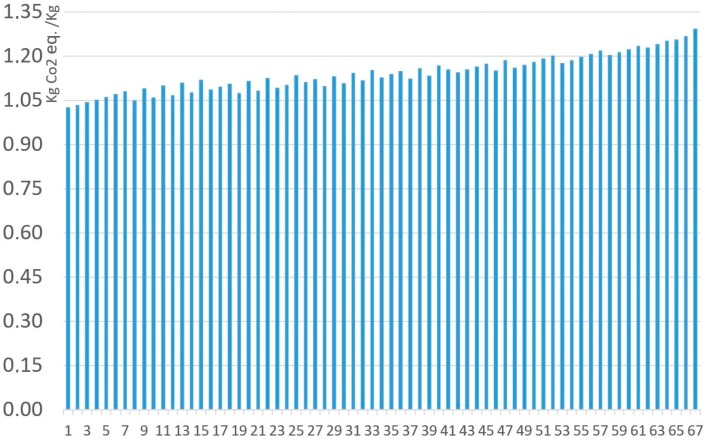
Environmental impact (RMA + Production) of 1 kg MnZn for the 67 ferrites, under the Carbon Footprint methodology.

Figure 7 and Figure 8 represent all the analyzed MnZn ferrite compositions, under the ReCiPe and Carbon Footprint methodologies. The 67 ferrites analyzed with the Monte Carlo analysis have been arranged from lowest to highest environmental impacts under ReCiPe, showing that the presence of manganese oxide represents the highest influence on the environmental impact. As shown in Table 8, environmental impact increases 652.3 mPt/kg and 0.267 kg CO_2_ eq. per kg when MnO content changes from 17% to 24.5% of the total. All the detailed data (composition, ReCiPe, and Carbon Footprint environmental impact, and the percentages of each material and the production processes for both methodologies) are shown in Table S1.

The results provided in Table S1 also allow us to analyze the relevance of the two considered LCA stages: Raw Material Acquisition and Production. Table 10 shows the percentages for both environmental impact methodologies.

As can be seen in the previous table, RMA environmental impact percentages have the highest contribution to the environmental impact, and increase as the overall impact does for both the ReCiPe and Carbon Footprint methodologies. Production processes represent between 0.78% and 1.11% percent under the ReCiPe methodology, showing the essential relevance of raw material consumption, especially of manganese, with a higher environmental impact. These production processes are more relevant under the Carbon Footprint methodology (19.61–24.71%), as it is highly influenced by the use of fossil fuels to generate electricity and heat. For both the analyzed methodologies, Raw Material Acquisition generates most of the impact; therefore, the composition is the key to determining the impact of MnZn ferrites.

**Figure 9:**
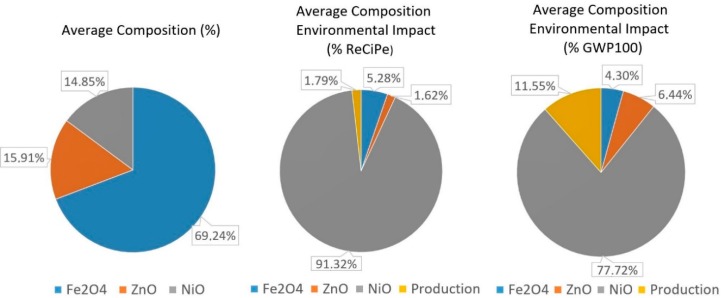
NiZn soft ferrite with average composition environmental impact in the ReCiPe and Carbon Footprint methodologies.

Figure 9 shows the composition and environmental impacts for the average NiZn soft ferrite. Analyzing how the environmental impact is created under the ReCiPe methodology allows us to conclude that the presence of nickel oxide causes almost 91.4% of the impact. Iron oxide generates 5.3% of it, while the production processes of this ferrite are below 1.8%. The use of zinc oxide produces the lowest environmental impact. Focusing on the environmental impact under the Carbon Footprint methodology, the content of NiO also generates the highest environmental impact: 77.7% of the total. In contrast, Fe_2_O_4_ with only 4.3% of the total environmental impact creates the lowest contribution to the environmental impact of the average composition. Finally, production processes account for 11.6%.

**Figure 10:**
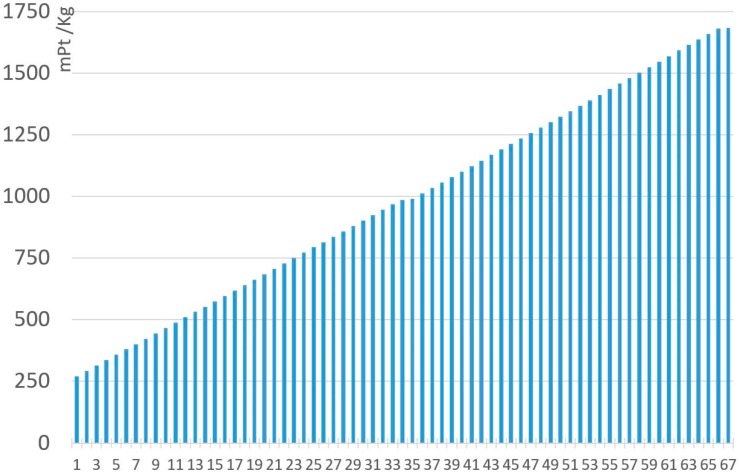
Environmental impact (RMA + Production). of 1 kg NiZn for the 67 ferrites, under the ReCiPe methodology.

**Figure 11:**
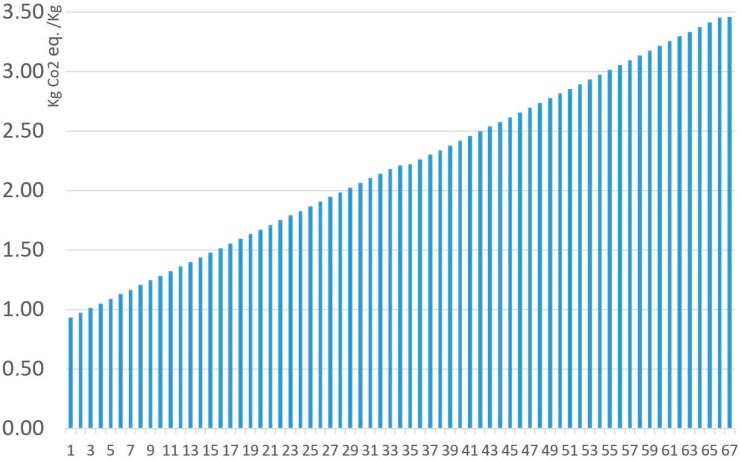
Environmental impact (RMA + Production) of 1 kg NiZn for the 67 ferrites, under the Carbon Footprint methodology.

Figure 10 and Figure 11 represent all the analyzed NiZn ferrite compositions, under the ReCiPe and Carbon Footprint methodologies. The 67 ferrites analyzed with the Monte Carlo analysis have also been arranged from lowest to highest environmental impacts under ReCiPe, showing that the presence of nickel oxide has the greatest influence on the environmental impact. Focusing on NiO content, an increase to the maximum composition value represents a rise of almost 1410 mPt/kg in the ReCiPe methodology, and a rise of 2.52 kg CO_2_ eq. per kg in the Carbon Footprint methodology. All the specific data are shown in Table S2.

The results provided in Table S2 also allow us to analyze the relevance of the two considered LCA stages: Raw Material Acquisition and Production. Table 11 shows in detail the percentages for both methodologies.

As can be seen in the previous table, RMA environmental impact percentages also make the highest contribution to the environmental impact, and their relevance increases as the overall impact does for both methodologies. Production processes represent between 1.03% and 6.43% under the ReCiPe methodology, again showing the high relevance of raw material consumption. This production percentage range is wider than the one calculated for MnZn ferrites, as NiZn ferrites also have a wide range of environmental impact results. This is mainly due to the presence of nickel, another material with a high environmental impact. These production processes are, again, more relevant under the Carbon Footprint methodology (7.33–27.14%).

For both methodologies, and for both ferrites, the Raw Material Acquisition generates most of the impact; therefore, the composition has been confirmed as the key to determining the impact of MnZn and NiZn ferrites.

**Figure 12:**
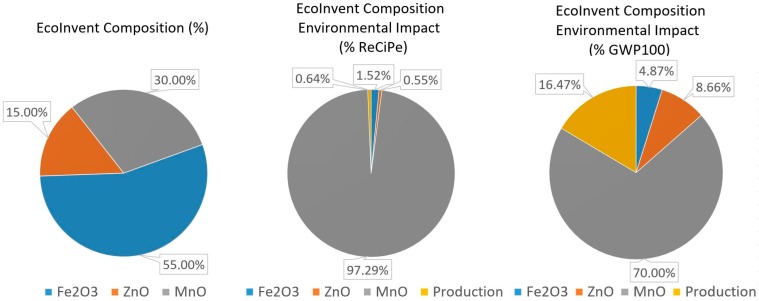
EcoInvent soft ferrite environmental impact in the ReCiPe and Carbon Footprint methodologies.

Table 12 shows the environmental results for the EcoInvent “Ferrite production {GLO}” dataset, used in this paper as a benchmark. The EcoInvent ferrite composition has a higher manganese content than the MnZn soft ferrites previously analyzed in this article and no nickel content. As previously explained, the presence of manganese generates a relevant environmental impact—in this case, 21.6% higher than the maximum MnZn ferrite examined in this article. Figure 12 shows that, as the EcoInvent ferrite composition has a high manganese oxide content (30%, higher than the composition ranges for MnZn soft ferrites), this presence also creates the highest environmental impact, almost 97.3% under ReCiPe and 70% under the Carbon Footprint. Nevertheless, the rest of the composition and the production processes are very low in ReCiPe. Under the Carbon Footprint methodology, production processes generate 16.5%, whereas ZnO and iron oxide content generates 8.7% and 4.9% respectively. 

These results show the relevance of accurately calculating the environmental impact of soft ferrites based on their composition. Although the RMA and production percentages tendencies are similar, the overall results in both categories present significant differences, especially for soft ferrites with low manganese or nickel content, which have a much lower environmental impact than the ones provided by EcoInvent.

The use of the EcoInvent dataset is valid when the consumption of soft ferrites is not relevant. However, it would lead to significant errors if it is used in products with a high presence of soft ferrites. These results are of interest for materials scientists, engineers, and LCA practitioners as they can help to better select soft ferrites, being able to calculate their environmental impact considering its composition, which is not currently possible using only the standard EcoInvent dataset.

## 4. Conclusions

This article shows the importance of considering material composition to accurately assess the environmental impact of MnZn and NiZn soft ferrites. To do that, the EcoInvent “Ferrite production {GLO}” has been used as a benchmark, customizing it to analyze the composition ranges of MnZn and NiZn soft ferrites. The EcoInvent v3.4 database was used to develop the life cycle inventory. The software used to perform the LCA was SimaPro 8.4, developed by Pré Consultants. The ReCiPe EndPoint (H/A) and IPCC 2013 Carbon footprint GWP100a have been used to perform the environmental impact study.

Both types of soft ferrites have been chosen as they share a common working area, which means that, for some applications, engineers can choose between both soft ferrite materials. This research will allow scientists and engineers to compare these ferrites based not only on cost and properties, but also on environmental impact, as this mainly depends on the composition, and varies significantly from the environmental results of the EcoInvent ferrite dataset.

For MnZn ferrites, the environmental impact calculated with the ReCiPe EndPoint methodology varies from 1572 mPt/kg to 2224 mPt/kg—a variation of 41.5%—whereas when using the Carbon Footprint methodology it varies 26%, from 1.025 to 1.292 kg CO_2_ eq. per kg. These values mainly depend on the total content of manganese, which can change from 17% to 24.5%. These values are also significantly different from the ones from the EcoInvent ferrite dataset: 2704 mPt/Kg and 1.54 kg CO_2_ eq. per kg.

In the case of NiZn soft ferrites, the environmental impact varies almost by 520% using the ReCiPe EndPoint methodology, from 272 up to 1682 mPt/kg and, around 270% in the Carbon Footprint methodology, from 0.94 up to 3.46 kg CO_2_ eq. per kg. NiZn values are always lower than the EcoInvent ferrite under the ReCiPe methodology, but may be lower or higher than the EcoInvent under the Carbon Footprint, depending on its nickel content. NiZn ferrite also shows a wider range of impact results, with this type of ferrite being more sensitive to the percentage of nickel content than MnZn ferrites are to the manganese content. These results are, therefore, of interest for materials scientists, engineers, and LCA practitioners, as they can help them with the selection of soft ferrites while considering the environmental impact.

The main factors that influence the results are the presence of manganese and nickel, respectively. They have a high environmental impact per kg in both methodologies and are also considered as strategic materials by the EU. In fact, the EU shows that their supply risk has been increasing over the last years, constituting a potential issue for European manufacturers.

For the analyzed soft ferrites, production processes are not a critical factor in the environmental impact. However, these production processes are much more relevant under Carbon Footprint than under the ReCiPe methodology, mainly due to their energy consumption (electricity, coal, and natural gas).

The article highlights the importance of considering exact material composition when calculating the environmental impact. Comparing both soft ferrite types under ReCiPe, most NiZn have a lower impact than MnZn. However, this conclusion is not valid when analyzed under Carbon Footprint. The EcoInvent ferrite dataset should only be used when ferrites are not relevant to the LCA, as more accurate calculations for MnZn and NiZn ferrites show that results can be 10 times lower than for EcoInvent (for the minimum impact of NiZn when using the ReCiPe methodology), or up to 124% higher (when analyzing the maximum impact of NiZn under Carbon Footprint).

## Figures and Tables

**Table 1:** EcoInvent dataset selection.

Material	Dataset
Iron Oxides	market for portafer—GLO
Zinc Oxide	market for zinc oxide—GLO
Manganese Oxide	market for manganese—GLO
Nickel Oxide	market for nickel—GLO

**Table 2:** Material molar composition percentage of MnZn soft ferrites.

Molar Percentage	Fe_2_O_3_	ZnO	MnO
Minimum (%)	50	10	30
Maximum (%)	60	30	40

**Table 3:** Material mass composition percentage of MnZn soft ferrites.

Mass Percentage	Fe_2_O_3_	ZnO	MnO
Minimum (%)	68.0	6.5	17.0
Maximum (%)	76.5	14.0	24.5

**Table 4:** Material molar composition percentage of NiZn soft ferrites.

Molar Percentage	Fe_2_O_4_	ZnO	NiO
Minimum (%)	50	10	5
Maximum (%)	50	90	45

**Table 5:** Material mass composition percentage of NiZn soft ferrites.

Mass Percentage	Fe_2_O_4_	ZnO	NiO
Minimum (%)	68.5	3.2	2.9
Maximum (%)	70.0	28.6	28.8

**Table 6:** Production processes from the EcoInvent inventory for soft ferrites.

Input	Quantity per kg
Production processes electricity consumption	0.01 kWh
Production processes heat (natural gas)	0.0363 MJ
Production processes heat (anthracite)	1.0064 MJ
Production processes heat (other sources)	0.4236 MJ
Infrastructure efforts	2.5 × 10^−11^ factories

**Table 7:** EcoInvent distances and mode of transportation from suppliers to the manufacturing plant.

Material	Mode of Transport	Kilometers
Iron Oxides	Truck	86
	Train	191
	Freight ship	5851
Nickel Oxide	Truck	361
	Train	345
	Freight ship	400
Zinc Oxide	Truck	209
	Train	309
	Freight ship	624
Manganese Oxide	Truck	361
	Train	345
	Freight ship	400

**Table 8:** Minimum, maximum, and average environmental impact of 1 kg MnZn soft ferrite (RMA + Production).

	Fe_2_O_3_ (%)	ZnO (%)	MnO (%)	ReCiPe (mPt/kg)	IPCC 2013 (kg CO_2_ eq.)
Minimum	76.5	6.5	17.0	1571.6	1.026
Average	70	10	20	1833.3	1.156
Maximum	68	7.5	24.5	2223.9	1.292

**Table 9:** Minimum, maximum, and average environmental impact of 1 kg NiZn soft ferrite (RMA + Production).

	Fe_2_O_4_ (%)	ZnO (%)	NiO (%)	ReCiPe (mPt/kg)	IPCC 2013 (kg CO_2_ eq.)
Minimum	68.5	28.6	2.9	271.0	0.934
Average	69	16	15	985.7	2.213
Maximum	70	3.2	26.8	1681.8	3.455

**Table 10:** RMA and production percentages for minimum, maximum, and average MnZn soft ferrites.

	Fe_2_O_3_ (%)	ZnO (%)	MnO (%)	ReCiPe RMA (%)	ReCiPe Production (%)	IPCC 2013 RMA (%)	IPCC 2013 Production (%)
Minimum	76.5	6.5	17.0	98.89	1.11	75.29	24.71
Average	70	10	20	99.05	0.95	78.07	21.93
Maximum	68	7.5	24.5	99.22	0.78	80.39	19.61

**Table 11:** RMA and production percentages for minimum, maximum, and average NiZn soft ferrite.

	Fe_2_O_4_ (%)	ZnO (%)	NiO (%)	ReCiPe RMA (%)	ReCiPe Production (%)	IPCC 2013 RMA (%)	IPCC 2013 Production (%)
Minimum	68.5	28.6	2.9	93.57	6.43	72.86	27.14
Average	69	16	15	98.23	1.77	88.55	11.45
Maximum	70	3.2	26.8	98.97	1.03	92.67	7.33

**Table 12:** EcoInvent ferrite dataset environmental impact in the ReCiPe and Carbon Footprint methodology.

Fe_2_O_3_ (%)	ZnO (%)	MnO (%)	ReCiPe (mPt/kg)	IPCC 2013 (kg CO_2_ eq.)
55.0	15.0	30.0	2704.0	1.5387

## Supplementary Materials

The following are available online at http://www.mdpi.com/1996-1944/11/10/1789/s1.
