# Supplementary material for: Influence of the Composition on the Environmental Impact of Soft Ferrites
Source: Materials (Basel). 2018 Sep 20;11(10):1789. doi: 10.3390/ma11101789 (PMC6213066; doi:10.3390/ma11101789)
Supplement: Supplementary file 1 [file materials-11-01789-s001.pdf]

Supplementary Materials: Influence of the Composition on the Environmental Impact of Soft Ferrites

Patricia Gómez, Daniel Elduque, Carmelo Pina and Carlos Javierre

Table S1. MnZn ferrites analysis (composition percentages, environmental impacts of both methodologies, and material and production percentages in both methodologies).

Composition (%)					ReCiPe (%)				IPCC 2013 GWP 100y (%)			
Fe_2O_3	ZnO	MnO	ReCiPe (mPts)	IPCC 2013 (Kg CO_2 eq.)	Fe_2O_3 RMA	ZnO RMA	MnO RMA	Production	Fe_2O_3 RMA	ZnO RMA	MnO RMA	Production
76.50%	6.50%	17.00%	1571.6	1.0257	3.63%	0.41%	94.85%	1.11%	10.16%	5.63%	59.51%	24.71%
75.70%	7.20%	17.10%	1580.5	1.0344	3.57%	0.45%	94.88%	1.10%	9.97%	6.18%	59.35%	24.50%
74.89%	7.90%	17.21%	1590.0	1.0435	3.51%	0.49%	94.90%	1.10%	9.77%	6.73%	59.21%	24.29%
74.07%	8.62%	17.32%	1599.7	1.0526	3.45%	0.53%	94.93%	1.09%	9.58%	7.27%	59.07%	24.08%
73.23%	9.34%	17.43%	1609.6	1.0619	3.39%	0.58%	94.95%	1.08%	9.39%	7.81%	58.93%	23.87%
72.39%	10.07%	17.54%	1619.5	1.0712	3.33%	0.62%	94.98%	1.08%	9.20%	8.35%	58.79%	23.66%
71.54%	10.81%	17.65%	1629.6	1.0808	3.27%	0.66%	95.00%	1.07%	9.01%	8.88%	58.65%	23.45%
75.77%	6.55%	17.68%	1631.1	1.0497	3.46%	0.40%	95.07%	1.07%	9.83%	5.54%	60.49%	24.14%
70.67%	11.56%	17.77%	1639.8	1.0904	3.21%	0.70%	95.03%	1.06%	8.83%	9.42%	58.51%	23.24%
74.95%	7.25%	17.80%	1641.1	1.0589	3.40%	0.44%	95.10%	1.06%	9.64%	6.08%	60.34%	23.94%
69.79%	12.32%	17.89%	1650.2	1.1001	3.15%	0.74%	95.05%	1.06%	8.64%	9.95%	58.38%	23.04%
74.13%	7.96%	17.91%	1651.1	1.0681	3.34%	0.48%	95.12%	1.06%	9.45%	6.62%	60.20%	23.73%
68.90%	13.09%	18.01%	1660.6	1.1100	3.09%	0.78%	95.08%	1.05%	8.45%	10.48%	58.24%	22.83%
73.30%	8.68%	18.02%	1661.3	1.0775	3.29%	0.52%	95.14%	1.05%	9.26%	7.15%	60.06%	23.52%
68.00%	13.87%	18.13%	1671.3	1.1200	3.03%	0.82%	95.10%	1.04%	8.27%	11.00%	58.10%	22.63%
72.45%	9.41%	18.14%	1671.6	1.0870	3.23%	0.56%	95.17%	1.04%	9.08%	7.69%	59.92%	23.32%
71.60%	10.14%	18.26%	1682.0	1.0966	3.17%	0.60%	95.19%	1.04%	8.89%	8.22%	59.78%	23.11%
70.73%	10.89%	18.38%	1692.5	1.1064	3.11%	0.64%	95.22%	1.03%	8.71%	8.74%	59.64%	22.91%

75.02%	6.60%	18.39%	1692.2	1.0743	3.30%	0.39%	95.28%	1.03%	9.51%	5.45%	61.45%	23.59%
69.85%	11.65%	18.50%	1703.3	1.1162	3.06%	0.68%	95.24%	1.02%	8.52%	9.27%	59.50%	22.71%
74.19%	7.30%	18.50%	1702.5	1.0837	3.25%	0.43%	95.30%	1.02%	9.32%	5.99%	61.30%	23.39%
68.96%	12.41%	18.62%	1714.1	1.1262	3.00%	0.72%	95.27%	1.02%	8.34%	9.79%	59.37%	22.50%
73.36%	8.02%	18.62%	1713.0	1.0932	3.19%	0.46%	95.33%	1.02%	9.14%	6.51%	61.16%	23.18%
72.52%	8.74%	18.74%	1723.7	1.1028	3.13%	0.50%	95.35%	1.01%	8.95%	7.04%	61.02%	22.98%
68.06%	13.19%	18.75%	1725.1	1.1364	2.94%	0.76%	95.29%	1.01%	8.16%	10.31%	59.23%	22.30%
71.66%	9.48%	18.86%	1734.5	1.1125	3.08%	0.54%	95.37%	1.01%	8.77%	7.57%	60.88%	22.78%
70.79%	10.22%	18.99%	1745.4	1.1224	3.02%	0.58%	95.40%	1.00%	8.59%	8.09%	60.74%	22.58%
74.26%	6.64%	19.10%	1754.1	1.0993	3.15%	0.38%	95.48%	0.99%	9.20%	5.37%	62.38%	23.06%
69.92%	10.97%	19.11%	1756.4	1.1323	2.97%	0.62%	95.42%	0.99%	8.41%	8.61%	60.60%	22.38%
73.42%	7.36%	19.22%	1764.9	1.1089	3.10%	0.41%	95.50%	0.99%	9.02%	5.89%	62.23%	22.86%
69.03%	11.73%	19.24%	1767.6	1.1425	2.91%	0.66%	95.45%	0.99%	8.23%	9.12%	60.46%	22.18%
72.58%	8.08%	19.35%	1775.9	1.1186	3.04%	0.45%	95.52%	0.98%	8.84%	6.41%	62.09%	22.66%
68.12%	12.51%	19.37%	1779.0	1.1527	2.85%	0.70%	95.47%	0.98%	8.05%	9.64%	60.33%	21.99%
71.72%	8.81%	19.47%	1787.0	1.1284	2.99%	0.49%	95.55%	0.98%	8.65%	6.93%	61.95%	22.46%
70.86%	9.55%	19.60%	1798.3	1.1384	2.94%	0.53%	95.57%	0.97%	8.48%	7.45%	61.81%	22.26%
69.98%	10.30%	19.73%	1809.7	1.1485	2.88%	0.56%	95.59%	0.96%	8.30%	7.96%	61.67%	22.07%
73.49%	6.69%	19.82%	1816.9	1.1246	3.01%	0.37%	95.66%	0.96%	8.90%	5.29%	63.28%	22.54%
69.09%	11.05%	19.86%	1821.3	1.1587	2.83%	0.60%	95.61%	0.96%	8.12%	8.47%	61.53%	21.87%
72.64%	7.41%	19.95%	1828.2	1.1344	2.96%	0.40%	95.68%	0.95%	8.72%	5.80%	63.14%	22.34%
68.18%	11.82%	19.99%	1833.0	1.1691	2.77%	0.64%	95.64%	0.95%	7.94%	8.98%	61.40%	21.68%
70.00%	10.00%	20.00%	1833.3	1.1557	2.84%	0.54%	95.66%	0.95%	8.25%	7.69%	62.14%	21.93%
71.79%	8.14%	20.08%	1839.7	1.1444	2.91%	0.44%	95.71%	0.95%	8.54%	6.31%	63.00%	22.15%
70.92%	8.87%	20.21%	1851.3	1.1545	2.85%	0.48%	95.73%	0.94%	8.36%	6.83%	62.85%	21.95%
70.04%	9.62%	20.34%	1863.1	1.1647	2.80%	0.51%	95.75%	0.94%	8.19%	7.33%	62.71%	21.76%
69.15%	10.37%	20.48%	1875.0	1.1750	2.75%	0.55%	95.77%	0.93%	8.01%	7.84%	62.58%	21.57%
72.70%	6.74%	20.55%	1880.6	1.1503	2.88%	0.36%	95.84%	0.93%	8.61%	5.21%	64.15%	22.03%

68.25%	11.14%	20.62%	1887.1	1.1855	2.69%	0.59%	95.80%	0.92%	7.84%	8.35%	62.44%	21.38%
71.85%	7.46%	20.69%	1892.4	1.1603	2.83%	0.39%	95.86%	0.92%	8.43%	5.71%	64.01%	21.84%
70.98%	8.20%	20.82%	1904.4	1.1705	2.78%	0.43%	95.88%	0.92%	8.26%	6.22%	63.87%	21.65%
70.10%	8.94%	20.96%	1916.6	1.1809	2.72%	0.46%	95.90%	0.91%	8.08%	6.72%	63.73%	21.46%
69.21%	9.69%	21.10%	1928.9	1.1913	2.67%	0.50%	95.92%	0.90%	7.91%	7.22%	63.59%	21.27%
68.31%	10.45%	21.24%	1941.3	1.2019	2.62%	0.53%	95.95%	0.90%	7.74%	7.72%	63.45%	21.09%
71.91%	6.79%	21.30%	1945.3	1.1763	2.75%	0.35%	96.00%	0.90%	8.32%	5.13%	65.00%	21.55%
71.04%	7.52%	21.44%	1957.6	1.1867	2.70%	0.38%	96.02%	0.89%	8.15%	5.63%	64.86%	21.36%
70.16%	8.26%	21.58%	1970.1	1.1971	2.65%	0.42%	96.05%	0.88%	7.98%	6.13%	64.72%	21.17%
69.27%	9.01%	21.72%	1982.8	1.2077	2.60%	0.45%	96.07%	0.88%	7.81%	6.62%	64.58%	20.99%
68.37%	9.76%	21.87%	1995.6	1.2184	2.55%	0.49%	96.09%	0.87%	7.64%	7.12%	64.44%	20.80%
71.11%	6.84%	22.05%	2010.9	1.2028	2.63%	0.34%	96.16%	0.87%	8.05%	5.05%	65.83%	21.07%
70.23%	7.58%	22.20%	2023.8	1.2134	2.59%	0.37%	96.18%	0.86%	7.88%	5.55%	65.68%	20.89%
69.33%	8.32%	22.35%	2036.8	1.2241	2.54%	0.41%	96.20%	0.86%	7.71%	6.04%	65.54%	20.71%
68.43%	9.07%	22.50%	2050.0	1.2349	2.49%	0.44%	96.22%	0.85%	7.55%	6.53%	65.40%	20.52%
70.29%	6.89%	22.82%	2077.5	1.2297	2.52%	0.33%	96.31%	0.84%	7.78%	4.98%	66.62%	20.61%
69.40%	7.63%	22.97%	2091.0	1.2405	2.47%	0.36%	96.33%	0.83%	7.62%	5.47%	66.48%	20.43%
68.49%	8.38%	23.12%	2104.5	1.2514	2.42%	0.40%	96.35%	0.83%	7.45%	5.95%	66.34%	20.25%
69.46%	6.95%	23.60%	2145.2	1.2569	2.41%	0.32%	96.45%	0.81%	7.52%	4.91%	67.40%	20.16%
68.55%	7.69%	23.75%	2159.1	1.2680	2.37%	0.35%	96.47%	0.81%	7.36%	5.39%	67.26%	19.99%
68.00%	7.50%	24.50%	2223.9	1.2923	2.28%	0.33%	96.60%	0.78%	7.17%	5.15%	68.07%	19.61%

Table S2. NiZn ferrites analysis (composition percentages, environmental impacts of both methodologies, and material and production percentages in both methodologies).

**Composition (%)**					**ReCiPe (%)**				**IPCC 2013 GWP 100y (%)**			
Fe_2O_4	ZnO	NiO	ReCiPe (mPts)	IPCC 2013 (Kg CO_2 eq.)	Fe_2O_4 RMA	ZnO RMA	NiO RMA	Production	Fe_2O_4 RMA	ZnO RMA	NiO RMA	Production
68.50%	28.60%	2.90%	271.0	0.9340	18.83%	10.48%	64.26%	6.43%	9.99%	27.20%	35.68%	27.14%
68.53%	28.19%	3.28%	293.5	0.9740	17.40%	9.54%	67.12%	5.94%	9.58%	25.71%	38.69%	26.02%
68.55%	27.80%	3.65%	315.0	1.0126	16.21%	8.76%	69.49%	5.53%	9.22%	24.39%	41.36%	25.03%
68.57%	27.42%	4.01%	336.7	1.0512	15.17%	8.09%	71.56%	5.18%	8.88%	23.17%	43.84%	24.11%
68.60%	27.03%	4.38%	358.3	1.0898	14.26%	7.49%	73.38%	4.87%	8.57%	22.03%	46.15%	23.26%
68.62%	26.64%	4.74%	379.9	1.1285	13.46%	6.96%	74.99%	4.59%	8.28%	20.97%	48.29%	22.46%
68.64%	26.25%	5.11%	401.6	1.1672	12.73%	6.49%	76.43%	4.34%	8.01%	19.98%	50.30%	21.71%
68.66%	25.86%	5.48%	423.2	1.2059	12.09%	6.07%	77.73%	4.12%	7.75%	19.05%	52.18%	21.02%
68.68%	25.47%	5.84%	444.9	1.2447	11.50%	5.68%	78.90%	3.92%	7.51%	18.18%	53.95%	20.36%
68.71%	25.08%	6.21%	466.6	1.2834	10.97%	5.34%	79.96%	3.74%	7.29%	17.36%	55.60%	19.75%
68.73%	24.69%	6.58%	488.3	1.3222	10.49%	5.02%	80.92%	3.57%	7.08%	16.59%	57.17%	19.17%
68.75%	24.30%	6.95%	510.0	1.3610	10.04%	4.73%	81.81%	3.42%	6.88%	15.86%	58.64%	18.62%
68.78%	23.91%	7.31%	531.7	1.3999	9.64%	4.46%	82.62%	3.28%	6.69%	15.17%	60.03%	18.11%
68.80%	23.52%	7.68%	553.5	1.4388	9.26%	4.22%	83.37%	3.15%	6.51%	14.52%	61.35%	17.62%
68.82%	23.13%	8.05%	575.2	1.4777	8.91%	3.99%	84.06%	3.03%	6.34%	13.90%	62.60%	17.15%
68.84%	22.74%	8.42%	597.0	1.5166	8.59%	3.78%	84.71%	2.92%	6.18%	13.32%	63.79%	16.71%
68.87%	22.35%	8.79%	618.8	1.5555	8.29%	3.59%	85.31%	2.82%	6.03%	12.76%	64.92%	16.29%
68.89%	21.95%	9.16%	640.6	1.5945	8.01%	3.40%	85.86%	2.72%	5.88%	12.23%	65.99%	15.90%
68.91%	21.56%	9.53%	662.4	1.6335	7.75%	3.23%	86.39%	2.63%	5.74%	11.72%	67.01%	15.52%
68.93%	21.17%	9.90%	684.3	1.6725	7.51%	3.07%	86.88%	2.55%	5.61%	11.24%	67.99%	15.15%
68.96%	20.78%	10.27%	706.1	1.7116	7.28%	2.92%	87.33%	2.47%	5.49%	10.78%	68.92%	14.81%

68.98%	20.38%	10.64%	728.0	1.7506	7.06%	2.78%	87.77%	2.39%	5.37%	10.34%	69.81%	14.48%
69.00%	19.99%	11.01%	749.8	1.7897	6.86%	2.65%	88.17%	2.32%	5.25%	9.92%	70.67%	14.16%
69.02%	19.60%	11.38%	771.7	1.8289	6.66%	2.52%	88.56%	2.26%	5.14%	9.52%	71.48%	13.86%
69.05%	19.20%	11.75%	793.6	1.8680	6.48%	2.40%	88.92%	2.20%	5.03%	9.13%	72.27%	13.57%
69.07%	18.81%	12.12%	815.5	1.9072	6.31%	2.29%	89.26%	2.14%	4.93%	8.76%	73.02%	13.29%
69.09%	18.42%	12.49%	837.5	1.9464	6.15%	2.18%	89.59%	2.08%	4.83%	8.40%	73.74%	13.02%
69.12%	18.02%	12.86%	859.4	1.9856	5.99%	2.08%	89.90%	2.03%	4.74%	8.06%	74.43%	12.76%
69.14%	17.63%	13.24%	881.4	2.0249	5.84%	1.99%	90.19%	1.98%	4.65%	7.73%	75.10%	12.52%
69.16%	17.23%	13.61%	903.4	2.0642	5.70%	1.89%	90.47%	1.93%	4.56%	7.42%	75.74%	12.28%
69.18%	16.84%	13.98%	925.4	2.1035	5.57%	1.81%	90.74%	1.88%	4.48%	7.11%	76.36%	12.05%
69.21%	16.44%	14.35%	947.4	2.1428	5.44%	1.72%	90.99%	1.84%	4.40%	6.82%	76.96%	11.83%
69.23%	16.05%	14.73%	969.4	2.1821	5.32%	1.64%	91.24%	1.80%	4.32%	6.53%	77.53%	11.61%
69.00%	16.00%	15.00%	985.7	2.2130	5.22%	1.61%	91.40%	1.77%	4.25%	6.42%	77.88%	11.45%
69.25%	15.65%	15.10%	991.4	2.2215	5.20%	1.57%	91.47%	1.76%	4.24%	6.26%	78.09%	11.41%
69.27%	15.25%	15.47%	1013.5	2.2609	5.09%	1.49%	91.69%	1.72%	4.17%	5.99%	78.63%	11.21%
69.30%	14.86%	15.85%	1035.5	2.3004	4.99%	1.42%	91.91%	1.68%	4.10%	5.74%	79.14%	11.02%
69.32%	14.46%	16.22%	1057.6	2.3398	4.88%	1.36%	92.11%	1.65%	4.03%	5.49%	79.64%	10.83%
69.34%	14.06%	16.59%	1079.7	2.3793	4.78%	1.29%	92.31%	1.61%	3.97%	5.25%	80.13%	10.65%
69.37%	13.67%	16.97%	1101.8	2.4188	4.69%	1.23%	92.50%	1.58%	3.91%	5.02%	80.60%	10.48%
69.39%	13.27%	17.34%	1123.9	2.4584	4.60%	1.17%	92.68%	1.55%	3.84%	4.79%	81.05%	10.31%
69.41%	12.87%	17.72%	1146.1	2.4980	4.51%	1.11%	92.85%	1.52%	3.78%	4.58%	81.49%	10.15%
69.44%	12.47%	18.09%	1168.2	2.5375	4.43%	1.06%	93.02%	1.49%	3.73%	4.37%	81.92%	9.99%
69.46%	12.07%	18.47%	1190.4	2.5772	4.35%	1.01%	93.18%	1.46%	3.67%	4.16%	82.33%	9.83%
69.48%	11.68%	18.84%	1212.6	2.6168	4.27%	0.96%	93.34%	1.44%	3.62%	3.96%	82.74%	9.69%
69.50%	11.28%	19.22%	1234.8	2.6565	4.19%	0.91%	93.49%	1.41%	3.56%	3.77%	83.13%	9.54%
69.53%	10.88%	19.59%	1257.0	2.6962	4.12%	0.86%	93.63%	1.39%	3.51%	3.58%	83.50%	9.40%
69.55%	10.48%	19.97%	1279.2	2.7359	4.05%	0.81%	93.77%	1.36%	3.46%	3.40%	83.87%	9.26%
69.57%	10.08%	20.35%	1301.4	2.7757	3.98%	0.77%	93.91%	1.34%	3.41%	3.23%	84.23%	9.13%

69.60%	9.68%	20.72%	1323.7	2.8154	3.92%	0.73%	94.04%	1.32%	3.37%	3.05%	84.58%	9.00%
69.62%	9.28%	21.10%	1346.0	2.8552	3.85%	0.68%	94.17%	1.30%	3.32%	2.89%	84.92%	8.88%
69.64%	8.88%	21.48%	1368.2	2.8951	3.79%	0.64%	94.29%	1.27%	3.28%	2.72%	85.25%	8.75%
69.67%	8.48%	21.86%	1390.5	2.9349	3.73%	0.61%	94.41%	1.25%	3.23%	2.57%	85.57%	8.64%
69.69%	8.08%	22.23%	1412.9	2.9748	3.67%	0.57%	94.52%	1.23%	3.19%	2.41%	85.88%	8.52%
69.71%	7.67%	22.61%	1435.2	3.0147	3.62%	0.53%	94.64%	1.21%	3.15%	2.26%	86.18%	8.41%
69.74%	7.27%	22.99%	1457.5	3.0547	3.56%	0.50%	94.74%	1.20%	3.11%	2.11%	86.48%	8.30%
69.76%	6.87%	23.37%	1479.9	3.0946	3.51%	0.46%	94.85%	1.18%	3.07%	1.97%	86.77%	8.19%
69.78%	6.47%	23.75%	1502.3	3.1346	3.46%	0.43%	94.95%	1.16%	3.03%	1.83%	87.05%	8.09%
69.81%	6.07%	24.13%	1524.6	3.1746	3.41%	0.40%	95.05%	1.14%	2.99%	1.70%	87.32%	7.98%
69.83%	5.66%	24.51%	1547.0	3.2147	3.36%	0.36%	95.15%	1.13%	2.96%	1.57%	87.59%	7.88%
69.85%	5.26%	24.89%	1569.5	3.2547	3.32%	0.33%	95.24%	1.11%	2.92%	1.44%	87.85%	7.79%
69.88%	4.86%	25.27%	1591.9	3.2948	3.27%	0.30%	95.33%	1.10%	2.89%	1.31%	88.11%	7.69%
69.90%	4.46%	25.65%	1614.3	3.3350	3.23%	0.27%	95.42%	1.08%	2.85%	1.19%	88.36%	7.60%
69.92%	4.05%	26.03%	1636.8	3.3751	3.18%	0.25%	95.51%	1.07%	2.82%	1.07%	88.60%	7.51%
69.95%	3.65%	26.41%	1659.3	3.4153	3.14%	0.22%	95.59%	1.05%	2.79%	0.95%	88.84%	7.42%
69.97%	3.24%	26.79%	1681.8	3.4555	3.10%	0.19%	95.67%	1.04%	2.76%	0.83%	89.07%	7.33%
70.00%	3.20%	26.80%	1682.5	3.4565	3.10%	0.19%	95.68%	1.03%	2.76%	0.82%	89.09%	7.33%
